# A National Survey of the Prevalence of Chronic Pain in Nursing Students and the Associated Factors*

**DOI:** 10.17533/udea.iee.v40n1e09

**Published:** 2022-03-30

**Authors:** Maryam Shaygan, Banafsheh Tehranineshat, Agrin Mohammadi, Zohreh Foruhi

**Affiliations:** 1 Associate Professor. Email: m2620.shaygan@gmail.com Community Based Psychiatric Care Research Center, School of Nursing and Midwifery, Shiraz University of Medical Sciences, Shiraz, Iran Community Based Psychiatric Care Research Center School of Nursing and Midwifery Shiraz University of Medical Sciences Shiraz Iran m2620.shaygan@gmail.com; 2 Assistant Professor. Email: tbanafsheh@yahoo.com. Department of Nursing, School of Nursing and Midwifery, Hormozgan University of Medical Sciences, Bandar Abbas, Iran. Department of Nursing School of Nursing and Midwifery Hormozgan University of Medical Sciences Bandar Abbas Iran tbanafsheh@yahoo.com; 3 Student. Email: agrinmohammadi@yahoo.com Student Research Committee. Shiraz University of Medical Sciences, Shiraz, Iran Shiraz University of Medical Sciences Shiraz Iran agrinmohammadi@yahoo.com; 4 Student. Email: z.foruhi@yahoo.com Student Research Committee. Shiraz University of Medical Sciences, Shiraz, Iran Shiraz University of Medical Sciences Shiraz Iran z.foruhi@yahoo.com

**Keywords:** students, nursing, chronic pain, cross-sectional studies., estudiantes de enfermería, dolor crónico, estudios transversales., estudantes de enfermagem, dor crônica, estudos transversais

## Abstract

**Objective.:**

To examine the prevalence of chronic pain and the associated factors among nursing students.

**Methods.:**

This study is a descriptive, cross-sectional. The subjects were 1684 nursing students who were selected from the universities of medical sciences in Iran via cluster sampling in 2019. Data were collected using a three-part questionnaire: a demographic characteristics survey, characteristics of chronic pain, and a pain scale.

**Results.:**

The majority of the students were female (62.1%) and single (87%).The mean age of the participants was 22.4±2.96 years. The results of data analysis showed that 30.2% of the students suffered from chronic pain. The areas which were most affected by pain were: head (31.24%), abdomen (11.98%), and the back (9.23%). 56.4% of the nursing students declared the origin of their pain to be unknown, 22.7% attributed their pain to migraine, and 6.48% reported spinal disorders to be the cause of their pain. There was a significant relationship between the students’ chronic pain and the variables of age (higher in the 29-and-above age group), marital status (higher in married subjects), and education (higher in postgraduates).

**Conclusion.:**

A relatively large number of nursing students suffer from chronic pains. Nursing schools should contribute to improving students' knowledge of chronic pain prevention and management.

## Resumen

**Objetivo.:**

Estimar la prevalencia del dolor crónico y sus factores asociados entre los estudiantes de enfermería.

**Métodos.:**

Estudio descriptivo de corte transversal. Participaron 1684 estudiantes de enfermería (1496 de pregrado y 188 de Maestría) de las universidades de ciencias médicas en Irán seleccionados mediante un muestreo por conglomerados. Los datos se recogieron a partir de un cuestionario dividido en tres partes: una encuesta de características demográficas, características del dolor crónico y una escala de dolor.

**Resultados.:**

La mayoría de los estudiantes fueron de sexo femenino (62.1%) y solteros (87%). La edad media fue de 22.4±2.9 años. Los resultados del análisis de los datos mostraron que el 30.2% de los estudiantes sufría dolor crónico. Las zonas más afectadas por el dolor fueron: la cabeza (31.24%), el abdomen (11.98%) y la espalda (9.23%). El origen del dolor fue más frecuentemente atribuido a la migraña (22.7%) y a los trastornos de la columna vertebral (6.5%). Un 56.4% declaró desconocer el origen de su dolor. Hubo una relación significativa entre el dolor crónico de los estudiantes y las variables: edad (mayor en el grupo de 29 años o más), estado civil (mayor en los casados), educación (mayor en los estudiantes de maestría).

**Conclusión.:**

Un número relativamente elevado de estudiantes de enfermería sufre de dolores crónicos. Las escuelas de enfermería deben contribuir en mejorar el conocimiento de ellos acerca de la prevención y manejo del dolor crónico.

## Resumo

**Objetivo.:**

Estimar a prevalência da dor crónica e seus fatores associados entre os estudantes de enfermagem.

**Métodos.:**

Estudo descritivo de corte transversal. Os participantes foram 1684 estudantes de enfermagem (1496 de graduação e 188 de Mestrado) das universidades de ciências médicas no Irã que foram selecionados através de uma amostragem por conglomerados. Os dados se recolheram mediante um questionário dividido em três partes: uma enquete de características demográficas, características da dor crónica e uma escala de dor.

**Resultados.:**

A maioria dos estudantes foram do sexo feminino (62.1%) e solteiros (87%). A idade média dos participantes foi de 22.4±2.9 anos. Os resultados da análise dos dados mostraram que 30.2% dos estudantes sofriam dor crónica. As zonas mais afetadas pela dor foram: a cabeça (31.24%), o abdômen (11.98%) e as costas (9.23%). A origem da dor foi mais frequentemente atribuída à enxaqueca (22.7%) e aos transtornos da coluna vertebral (6.5%). Um 56.4% dos estudantes de enfermagem declararam desconhecer a origem da sua dor. Houve uma relação significativa entre a dor crónica dos estudantes e as variáveis: idade (maior no grupo de 29 anos ou mais), estado civil (maior nos casados), educação (maior nos estudantes de mestrado).

**Conclusão.:**

Um número relativamente elevado de estudantes de enfermagem sofre de dores crónicas. As escolas de enfermagem devem contribuir em melhorar o conhecimento dos estudantes sobre a prevenção e manejo da dor crónica.

## Introduction

Chronic pain is a prevalent healthcare issue which can affect all age groups and cause varying degrees of disability in individuals.(1) Chronic pain defines as persistent or recurrent pain that lasts longer than three months and is associated with several biological changes, as well as having some psychological and social elements contributing to its onset, maintenance, and exacerbation.(2) Pain has emerged as a prevalent health issue among college students. Many students are living with unrelieved pains which result in their low quality of life, poor academic performance, absenteeism, sleep disorder, and reduced psycho-social well-being.(3) Due to the financial burden which it imposes on the society and the healthcare system, the prevalence of chronic pain has emerged as a global issue with adverse effects on individuals’ daily activities. Even though young college students are among the main groups which suffer from chronic pain, few researchers have studied the prevalence and consequences of chronic pain among young adults and college students.(4) The results of some studies verify the high prevalence of musculoskeletal pain among college students: 42.8% of medical and non-health related students complain of neck pain and 72.1% of medical students complain of back pain.(1,5)

Despite the special lifestyle of healthcare personnel and college students, few studies have addressed musculoskeletal pain in this population.(6) In their cross-sectional study, Silva *et al.* report that 35.69% of the students of various medical specialties at Tauba University suffer from chronic pain.(7) Several studies in Iran have reported the prevalence of back pain and general pain in the adult population to be high; however, certain nursing-related activities, e.g. moving patients, and disregard for ergonomic principles cause nurses to be more prone to chronic pain and the physical limitations ensuing from it than others.(8,9) Engagement in nursing-related activities starts from nursing school (7) as nursing students are among the primary caregivers in Iran.

A review of the available literature shows that few studies have investigated the prevalence of chronic pain among nursing students.(4) Thus, there is a lack of precise information in this area. As nursing students are more prone to chronic pain than the other members of the society, study of chronic pain in this population is important. In addition, nursing students’ rigorous education and training can aggravate their chronic pain. Chronic pain can adversely affect the daily and academic activities of this young population, as well as their important decisions in their life and profession.(10) Study of chronic pain in its early stages and the factors related to it can help identify students who are at higher risk and contribute to development of more effective preventive programs. One of the most important measures in pain control is determining the number of individuals who suffer from pain and the causes of their pain.(11) As the physical and psychological well-being of nursing students plays a key part in the public health of the country in the future, the present study addresses the prevalence of different types of chronic pain and the factors associated with it in the nursing students of the universities of Iran. The aim of this study was to examine the prevalence of chronic pain and the associated factors among nursing students.

## Methods

This descriptive, cross-sectional study was conducted on the nursing students at the universities of medical sciences of Iran between February and October 2019. Based on the findings of previous studies and the assumption that at least 20% of nursing students suffer from chronic pain(11) and using a sample size formula and an attrition rate of 20%, sample size was set at a minimum of 1500 students. The inclusion criteria were students who finished at least one semester of study at the time at which they completed the questionnaire, students who did not work in the nursing profession or any other job, being willing to participate in the study and being a nursing student at one of the universities of medical sciences in Iran. The students who had a self-declared chronic physical condition not related to their pain (e.g. M.S, diabetes, cancer, congenital and developmental diseases, history of accidents, and surgery) were excluded.

After obtaining permission from the authorities at the nursing schools, the researchers used the lists of all the public nursing schools in the 5 geographic regions of Iran (north, south, east, west, and center). Initially, nursing schools were selected via cluster-random sampling: one school was simple randomly selected from each geographic region. Using a list of the names of all the undergraduate nursing students, the researchers coded the names and then the sample was selected from a random number table. The students who met the inclusion criteria were selected as potential participants. Next, the faculties were contacted and asked to submit the students’ class schedules. On the days when the selected students were present in school, the researcher met them and informed them about the objectives of the study and the definition of chronic pain. The questionnaires were completed by the students themselves. Data were collected over a 9-month period. When the questionnaires were being completed, one of the researchers (AM or ZF) was present to answer any queries.

30 questionnaires which had not been filled out completely were eliminated. Eventually, 1684 questionnaires (completed by 1045 female and 639 male nursing students) were considered for data analysis. Data were collected using a three-part questionnaire: a demographic characteristics survey, characteristics of chronic pain, and a pain scale. The demographic characteristics survey consisted of questions about age, gender, marital status, education, parents’ education, and place of residence. The section on characteristics of chronic pain addressed the location, severity, frequency, and cause of pain. To assess the severity of each student’s pain, the researchers used a pain visual analog scale, which is a 10-centimeter long vertical or horizontal line on which 0 represents absence of any pain and 10 represents extreme or unbearable pain. The concurrent validity of the scale compared to NPRS (numerical pain rating scale) is 0.71-0.78, and the test-retest reliability of the scale has been reported to be 0.71-0.99. (12) In Iran, the reliability of the scale has been reported to be 0.81.(9) The collected data were analyzed in SPSS v. 24 using descriptive and inferential (chi-square) statistics. Significance level was set at less than 0.05. The present study has been approved by the ethics committee of Shiraz University of Medical Sciences, Iran (IR.SUMS.REC.1397.052). All the nursing students were informed about the objectives of the study and asked to complete the informed consent form. The students were assured that their information would remain anonymous and confidential.

## Results

The present study was conducted on1496 undergraduate and 188 postgraduate nursing students. The majority of the students were found to be female (62.1%), between 18-28 years old (95%) and single (87%). The mean age of the students was 22.4±2.96 years and their grade point average was 16.03±1.18. (Table 1) Most of the students (88.8%) were bachelor’s degree candidates. The highest academic degree of the students’ fathers and mothers was high school diploma 778 (46.4%) and 718 (42.8%) respectively. The majority of the students 979 (58.1%) lived in a dormitory. 

**Table 1:** Descriptive statistics for demographic characteristics of 1684 nursing students

Characteristic	No.	%
Sex		
Male	639	37.9
Female	1045	62.1
Age		
18-28 years	1613	95.8
29-39 years	65	3.9
≥40 years	6	0.4
Marital status		
Single	1465	87
Married	219	13
Education		
Undergraduate	1496	88.8
Postgraduate	188	11.2
Father education *		
High school or less	404	24
Diploma	778	46.4
University degree	496	12.6
Mother education*		
High school or less	511	30.4
Diploma	718	42.8
University degree	449	26.8
Residence		
Living in campus	979	58.1
Living with the family	669	39.7
Other	36	2.1

Note: Sample size varies due to non-responses (*n=1678, missing=6).

In the present study, the prevalence of chronic pain in nursing students was found to be 30.2%. The most common areas which were affected by pain were, in descending order, the head (31.24%), the abdomen (11.98%), and the back (9.23%). 381 of the 509 students with chronic pain (74.8%) declared the severity of their pain to be grade 3 or higher, and the mean was 10.69±11.42. Most of the students (56.40%) reported the cause of their pain to be unknown, i.e. the cause of their chronic pain was not a definite pathological source, 117 (22.7%) attributed their pain to migraine, and 33 (6.48%) reported spinal disorders to be the cause of their pain (Table 2). Most of the students 151 (29.66%) reported the frequency of their chronic pain to be daily, and a few 14 (2.75%) reported their pain was permanent. 391 (76.80%) had not missed classes because of their chronic pain, but 81 (15.9%) had had delays which they attributed to their pain.

**Table 2:** Pain characteristics of nursing students (*n*=509)

Characteristic	No.	%
Pain location		
Head	159	31.24
Neck	36	7.07
Ear	10	1.96
Eye	43	8.45
Face	32	6.28
Shoulder	23	4.52
Wrist, hand, arm	25	4.91
Abdomen	61	11.98
Knee	40	7.86
Ankle, Leg	20	3.93
Back, waist	47	9.23
Chest	13	2.57
Pain intensity		
1	22	4.32
2	106	20.82
3	150	29.47
4	103	20.24
5	84	16.50
6	27	5.32
7	11	2.16
8	5	0.98
9	0	0
10	1	0.19
Cause pain		
Arthritis	18	3.54
Congenital	5	0.98
Neuropathy	5	0.98
Spinal cord	33	6.48
Injury	26	5.11
Surgery	2	0.39
Poor blood circulation	2	0.39
Migraine	117	22.98
Dystrophy	2	0.39
Osteoporosis	6	1.18
Multiple sclerosis	3	0.59
Lupus	3	0.59
Unknown	287	56.40
Frequency of chronic pain		
Permanent	14	2.75
Daily	151	29.66
Weekly	224	44.01
monthly	120	23.58
University absence due to pain		
No absence	391	76.80
Absence	118	23.20
Delay due to pain		
Yes	81	15.90
No	428	84.10

The results showed that there was a significant relationship between the students’ pain and their age (higher in the 29-and-above age group), marital status (higher in married students), education (higher in postgraduates). However, the relationship between pain and the variables of gender, father’s education, mother’s education, place of residence, and grade point average were not significant. (Table 3)

**Table 3:** Factors associated with chronic pain (*n*=1684)

**Pain** **Variables**	No pain *n* (%)	Chronic pain *n* (%)
Gender		
Female (*n*=1045)	719 (68.8)	326 (31.2)
Male (*n*=639)	456 (71.3)	183 (28.7)
Age		
18-28 years (n=1613)	1136 (70.4)	477 (29.6)
29-39 years (n=65)	36 (55.4)	29 (44.6)
≥40 years (n=6)	3 (50)	3 (50)
Marital status		
Single (n=1465)	1041 (71.1)	424 (28.9)
Married (n=219)	134 (61.2)	85 (38.8)
Education		
Undergraduate (n=1496)	1057 (70.6)	439 (29.4)
Postgraduate (n=188)	118 (62.8)	70 (37.2)
Father education		
High school or less (n=404)	276 (16.4)	128 (7.6)
Diploma (n=778)	539 (32.1)	239 (14.2)
University degree (n=496)	354 (21.1)	142 (8.5)
Mother education		
High school or less (n=511)	335 (20)	176 (10.5)
Diploma (n=718)	500 (29.8)	218 (13)
University degree (n=449)	334 (19.9)	115 (6.9)
Residence		
Living in campus (n=979)	696 (41.3)	283 (16.8)
Living (with family) (n=669)	450 (26.7)	219 (13)
Other (n=36)	29 (1.7)	7 (0.4)
Grade point average		
10-15 (n=640)	454 (27)	186 (11)
16-20 (n=1044)	721 (42.8)	323 (19.2)

## Discussion

In the present study, the prevalence of chronic pain in nursing students was found to be 30.2%. The most common areas which were affected by pain were, in descending order, the head, the abdomen, and the back. Most of the students reported the cause of their chronic pain to be unknown. Kodana *et al.,* report the prevalence of chronic pain in nursing students to be 79.2%.(3) A study of medical students reports that 88.5% of the subjects had suffered from musculoskeletal pain in at least one location (13). In the study of Abledu *et al*., 70.1% of the nursing students had suffered from musculoskeletal disorders in the past 12 months and 56.1% had been affected by the incapacitating consequences of pain. 44.6% of the students complained of pain in their necks, backs, lower backs, and wrists.(14) In a study of the prevalence of chronic pain in 1011 adults in Brazil, the results showed that the source of pain in 15% of the population was unknown. In descending order, the body parts most affected by pain were the upper parts, heads and necks, and lower parts.(15) Differences in the prevalence and locations of chronic pain can be attributed to differences in the study populations (race and ethnicity), designs, sample sizes, and other disorders and contributory factors. Moreover, stress and academic overload, sitting in classroom chairs for long periods, and poor sitting posture while studying are among the risk factors in college students’ musculoskeletal disorders (16)

The results of the present study showed that the highest frequency of chronic pain among nursing students belonged to the 29 and over age group and that there was a significant relationship between the students’ chronic pain and age. According to the study of Houde *et al.*, there is a significant positive relationship between pain and disability on the one hand and age on the other in individuals with back pain. The relationship is more significant in the youth than in the elderly.(17) However, in the study of UCEL and TORUN, where the majority of the participants were college students under 30, the relationship between the students’ age and perceived severity of pain was found to be insignificant.(18) Studying the prevalence of chronic back pain among nurses in Jordan, Alhadidi *et al*., report that 82.5% of nurses aged 35 suffer from chronic back pain.(19) However, inconsistency in results can be due to selection of sample, study settings and variation in the area and population of different countries. Also, the inconsistency between these research results may be due to the researchers’ use of different pain scales. Yet, the findings of studies confirm the increasing prevalence of chronic pain in various populations and age groups.(4) Chronic pain is not limited to older adults; Mill’s review study shows that chronic pain is prevalent among adolescents and young adults.(20) Moreover, it has been found that the elderly gradually adapts to performing their daily activities despite their chronic pain.(21)

The findings of the present study show that there is a significant relationship between pain and marital status. The results of the study of Mawdsley *et al.,* in England show that the presence of patients’ spouses in chronic pain management programs increases patients’ satisfaction and improves their perception of self-management behaviors and communication skills.(22) In their study of marital relationships and psychological resilience in patients with chronic pain in the U.S., Wade et al., report that the subjects who had lost their spouses felt less alienation, fear, anger, and depression than the other (married, divorced, and single) subjects. Satisfactory marital relationships help patients tolerate their disease more easily and feel less incapacitated.(23) On the other hand, poor marital adjustment or singlehood can increase stress and make patients disinclined to exercise health behaviors, including seeking appropriate medical care, which will aggravate their pain and suffering.(20)

The findings of the present study showed that there was a significant relationship between the nursing students’ education and chronic pain. Most of the nursing students in the present study were undergraduates who attributed their chronic pain to unknown causes, migraine, and spinal disorders, in descending order. In the study of Vujcic *et al*., the prevalence of back pain in medical students in the 5^th^ semester of their studies and above was higher than in others. The researchers mention the participation of medical students of higher years in practical training, their poor posture, and failure to exercise as the reasons for the higher incidence of pain in this group.(24) According to a study, musculoskeletal pain in medical students correlates with their clinical activities, the number of hours they use computers, history of trauma, family health history, and BMI. (25) In Iran, clinical training courses for undergraduate and postgraduate nursing students start from their second semester. During their clinical raining, nursing students should care for their patients, which entails remaining standing for long periods. Also, at school, they have to spend long hours sitting while attending classes or studying. Poor posture during clinical practice and long hours of sitting in classes and libraries can account for the occurrence of musculoskeletal pain in nursing students.

One of the limitations of the present study is that personal differences, e.g. different physical, emotional, psychological, and family conditions, between the nursing students may have affected the findings of the study. In addition, the fact that few studies have addressed chronic pain in nursing students and the factors associated with it restricted the possibility of comparing the findings of the present study with those of other similar studies. Another limitation of the present study is its use of a self-report questionnaire: it was possible that the nursing students would not provide honest answers. To minimize the impact of response bias, the researchers informed the participants about the objectives and applications of the study and distributed the questionnaires at appropriate times and places by prior arrangement with the students. Also, in the present study, the collected data did not include the participants’ BMIs, tobacco use, or exercise habits. Therefore, it is suggested that future studies include these variables. Also, one school was randomly selected from each geographic region through sampling; it would have been better if two or more schools had been selected.

## Conclusion

The findings of the study show that, though they are a young population, nursing students are at risk of suffering chronic pain and the negative consequences associated with it. It is, therefore, necessary that nursing students be educated about the contributing factors in chronic pain, good posture for studying, and physical activities. There is also need for educational programs to raise students’ awareness of the impact of pain on their mental health. Equipping dormitories with proper facilities, e.g. massage chairs, and providing counseling services for students who suffer from chronic pain can improve the physical and mental health of the students. These findings can help university authorities take the necessary measures toward prevention and management of pain in students who suffer from chronic pain, thereby helping the students’ academic performance and success. It is suggested that more studies be conducted in other countries to investigate the factors which contribute to nursing students’ chronic pain to add to the existing knowledge.
